# Direct determination of diploid genome sequences

**DOI:** 10.1101/gr.214874.116

**Published:** 2017-05

**Authors:** Neil I. Weisenfeld, Vijay Kumar, Preyas Shah, Deanna M. Church, David B. Jaffe

**Affiliations:** 10x Genomics, Pleasanton, California 94566, USA

## Abstract

Determining the genome sequence of an organism is challenging, yet fundamental to understanding its biology. Over the past decade, thousands of human genomes have been sequenced, contributing deeply to biomedical research. In the vast majority of cases, these have been analyzed by aligning sequence reads to a single reference genome, biasing the resulting analyses, and in general, failing to capture sequences novel to a given genome. Some de novo assemblies have been constructed free of reference bias, but nearly all were constructed by merging homologous loci into single “consensus” sequences, generally absent from nature. These assemblies do not correctly represent the diploid biology of an individual. In exactly two cases, true diploid de novo assemblies have been made, at great expense. One was generated using Sanger sequencing, and one using thousands of clone pools. Here, we demonstrate a straightforward and low-cost method for creating true diploid de novo assemblies. We make a single library from ∼1 ng of high molecular weight DNA, using the 10x Genomics microfluidic platform to partition the genome. We applied this technique to seven human samples, generating low-cost HiSeq X data, then assembled these using a new “pushbutton” algorithm, Supernova. Each computation took 2 d on a single server. Each yielded contigs longer than 100 kb, phase blocks longer than 2.5 Mb, and scaffolds longer than 15 Mb. Our method provides a scalable capability for determining the actual diploid genome sequence in a sample, opening the door to new approaches in genomic biology and medicine.

Determining the genome sequence of an individual organism is of fundamental importance to biology and medicine. Although the ability to correlate sequence with specific phenotypes has improved our understanding of human disease, the molecular basis of ∼20% of Mendelian phenotypes is still unknown (http://omim.org/statistics/geneMap), and the situation for common disease is much worse. Contributing to this is the incomplete elucidation of the genomic architecture of the genomes under study (Eichler et al. 2010).

Decades of research have yielded a vast array of laboratory and computational approaches directed at the problem of knowing the genome sequence of a given sample. These vary dramatically in their aggregate experimental burden, including input DNA amount, organizational complexity, laboratory and computational requirements for expertise and hardware, project complexity, cost and timeline, with greater burden tending to yield a higher quality genome sequence.

At the low end, and by far the most widely executed, are “resequencing” methods that generate short reads, then align them to a haploid reference sequence from the same species, to identify differences with it, thereby partially inferring the sequence of the sample (Li et al. 2008; McKenna et al. 2010). Several projects have generated and analyzed over a thousand human samples each, yielding extraordinarily deep information across populations (The 1000 Genomes Project Consortium 2015; Gudbjartsson et al. 2015; Nagasaki et al. 2015); although in general, such methods cannot completely catalog large-scale changes, nor distinguish between parental alleles. Moreover, such methods are intrinsically biased by comparison to a reference sequence, thus limiting their ability to see sequences in a sample that are significantly different from it (Chaisson et al. 2015b).

In contrast, an analysis of an individual genome would ideally start by reconstructing the genome sequence of the sample, without using a reference sequence. This de novo assembly process is difficult for large and complex genomes (Istrail et al. 2004; Chaisson et al. 2015a; Gordon et al. 2016; Steinberg et al. 2016). A core challenge is the correct representation of highly similar sequences, which range in scale from single base repeats (homopolymers) to large complex events including segmental duplications (Bailey et al. 2002).

There is an even larger scale at which similar sequences appear: homologous chromosomes, which are “repeats” across their entire extent. To correctly understand the biology of a diploid organism, these homologous chromosomes need to be separately represented (or phased), at least at the scale of genes (Muers 2011; Tewhey et al. 2011; Glusman et al. 2014; Snyder et al. 2015). This is required to correctly understand allele-specific expression and compound heterozygosity. For example, two frameshifts in one gene allele could have a completely different phenotype than one each in both alleles; likewise, larger-scale effects such as changes to gene copy number (Horton et al. 2008; Pyo et al. 2010) need to be understood separately for each homologous chromosome. However, precisely because homologous chromosomes are so similar, it is challenging to keep them separate in assemblies.

In fact, the standard of the field for genome assembly has been to represent homologous loci by a single haploid “consensus” sequence that merges parental chromosomes. This loses “half” of the information, and in general does not represent a true physical sequence present in nature. As a step in the right direction, one could generate a haploid assembly together with a phased catalog of differences between the two originating chromosomes (Pendleton et al. 2015; Mostovoy et al. 2016). However, for the same reasons that a de novo assembly can carry more information than a simple catalog of differences with a reference sequence, these “phased haploid” assemblies carry less information than true diploid assemblies that separately display homologous loci. In a few cases, these diploid de novo assemblies have been demonstrated for small and midsized genomes (Jones et al. 2004; Chin et al. 2016). There are two extant instances of diploid de novo assemblies of human genomes—one obtained by Sanger sequencing of multiple libraries (Levy et al. 2007), and one from thousands of separate clone pools, each representing a small, low-throughput partition of the genome (Cao et al. 2015).

In this work, we bridge the gap between low-cost resequencing approaches and high-cost diploid assembly approaches, by creating diploid de novo assemblies, at very low experimental burden. Our method is also based on genome partitioning. Using an automated microfluidic system (Zheng et al. 2016), we are able to generate the entirety of data for an assembly project from one library. Moreover, this library is made from ∼1 ng high molecular weight DNA, far less than alternative approaches. The cost of our data is in the range of low end methods based on read alignment, and special expertise is not required for assembly, because the process is automatic.

To demonstrate our method, we chose seven human samples from diverse populations, including four for which parental data was available, allowing us to test phasing accuracy. This set also included a sample for which 340 Mb of finished sequence was generated during the Human Genome Project (HGP), thus providing an unprecedented source of truth data, and allowing us to assess the fine-scale accuracy of our method in a way that has not been previously possible. We assembled these samples using a new algorithm called Supernova Assembler. Both the laboratory and computational methods are encapsulated in a complete commercial system from 10x Genomics. Open source software, data sets, and assemblies described in this work are publicly available.

## Results

### Data generation

We provide a conceptual explanation of our schema for data generation, including the fundamental characteristics of the data type. Our method uses an updated version of the 10x microfluidic gel bead partitioning system (Zheng et al. 2016), called the Chromium Genome Reagent Kit (Methods). Library construction starts from 1.25 ng of DNA having size 50 kb or longer (Zhang et al. 2012), from a single individual organism or clonal population (such as a cell line).

Briefly, the system exploits a reagent consisting of several million gel beads, with each bead containing many copies of a 16-base barcode unique to that bead. A microfluidic device delivers individual beads into approximately one million partitions, along with high-molecular weight genomic DNA molecules and reagents. Each partition receives several long molecules (as discussed below), and the molecular biology of the system is arranged to create constructs having the barcode, along with ∼350 bp of genomic DNA from a molecule, sandwiched between Illumina adapters. The barcode is placed at the beginning of the first read in a pair. These constructs are then sequenced on an Illumina instrument, yielding groups of read pairs organized by barcode. Sets of these read pairs that originate from the same molecule are called Linked-Reads.

We describe the sequencing configuration that we used for de novo assembly. Paired reads of length 150 bases each are generated. This read length was chosen so that data could be sequenced on the HiSeq X instrument, which yields the lowest cost data among Illumina instruments and that has a maximum read length of 150. Data can also be generated on the HiSeq 2500 in rapid run mode. We tested the HiSeq 4000, observing a twofold reduction in contig size (Supplemental Note 1). We recommend that samples be sequenced to 56× (including bases in barcodes), or about 1200 M reads for a human genome. Lower coverage is possible and described later.

Here, we describe the model behavior of the system. Of the DNA that is loaded, ∼40% makes it completely through the process and contributes to the sequencing library. For 1.25 ng of loaded material, distributed across 10^6^ partitions and supposing that all molecules had size 50 kb, the mean number of molecules per partition would be about 10, in total representing ∼0.5 Mb of the genome per partition. At 56× coverage, the mean number of Linked-Reads (read pairs) per molecule for a human genome would thus be (1200M/2)/(10^6^ × 10) = 60, and covering the molecule to depth (120 · 150)/(50,000) = 0.36×. Importantly, the mode of operation of the system is to provide shallow coverage by many reads for each of many long molecules. In particular the system is not designed to deeply cover individual molecules, in contrast to Synthetic Long-Reads (Voskoboynik et al. 2013).

For smaller genomes, assuming that the same DNA mass was loaded and that the library was sequenced to the same read depth, the number of Linked-Reads (read pairs) per molecule would drop proportionally, which would reduce the power of the data type. For example, for a genome whose size is one-tenth the size of the human genome (320 Mb), the mean number of Linked-Reads per molecule would be about six, and the distance between Linked-Reads would be about 8 kb, making it hard to anchor barcodes to short initial contigs. Modifications to workflow, such as loading less DNA and/or increasing coverage would be potential solutions for smaller genomes, but are not described here.

Using the method described above, we generated data sets from seven human individuals of varied ancestry and sex (Table 1). All were created from DNA of size > 90 kb, measured by length-weighted mean (Table 1). We tested the performance of the system on DNA of several different sizes, noting degradation in performance (Supplemental Note 2), particularly for DNA <30 kb (DNA of size ∼20 kb yielded scaffolds of N50 size 0.6 Mb, whereas DNA of size ∼50 kb yielded scaffolds of N50 size 12.8 Mb). We also tested the effect of reducing coverage from 56× to 38× (Supplemental Note 3), noting some degradation in quality, e.g., scaffold N50 declining from 17 to 12 Mb.

**Table 1.**
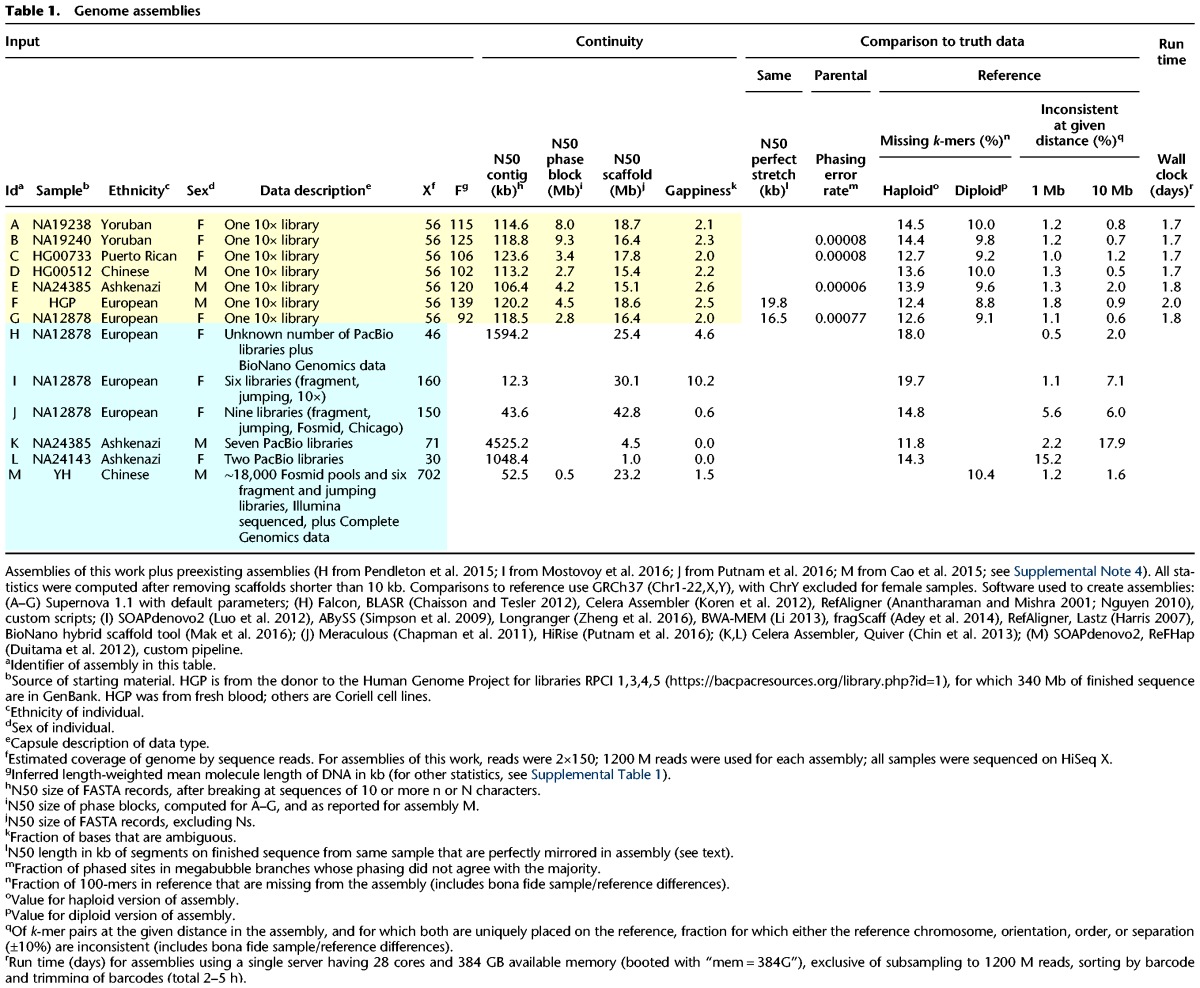
Genome assemblies

### De novo assembly

Because barcoded 10x data provide shallow coverage of each molecule, it is not possible to separately assemble the reads from each barcoded partition, which would otherwise be a natural approach (Voskoboynik et al. 2013). Instead the assembly process creates progressively larger contigs. At the point where many contigs are at least a few kb long, most molecules that “pass through” a given contig will have at least one read landing on it. This information about partitions touching the contig may be used to link to other contigs; moreover, all reads from all partitions touching the contig may be assembled together. This is the Supernova analog of single-partition assembly.

Following this strategy, barcodes play a relatively minor role in the initial process. To start out, we use a de Bruijn graph approach (Pevzner et al. 2001), adapting the method of DISCOVAR (Weisenfeld et al. 2014). *k*-mers (*k* = 48) are prefiltered to remove those present in only one barcode, thus reducing the incidence of false *k*-mers, i.e., those absent from the sample. The remaining *k*-mers are formed into an initial directed graph, in which edges represent unbranched DNA sequences, and abutting edges overlap by *k*−1 bases. Operations are then carried out to recover missing *k*-mers and remove residual false *k*-mers (Weisenfeld et al. 2014). At this point, the graph (called the base graph) is an approximation to what would be obtained by collapsing the true sample genome sequence along identical 48-base sequences (Butler et al. 2008). We then use the read pairs to effectively increase *k* to about 200, so that the new graph represents an approximation to what would be obtained by collapsing the true sample genome sequence along identical 200-base sequences, thus achieving considerably greater resolution (Methods).

**Figure 1.**
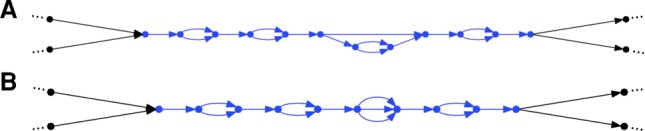
Lines in an assembly graph. Each edge represents a DNA sequence. (*A*) Blue portion describes a line in an assembly graph, which is an acyclic graph part bounded on both ends by single edges. The line alternates between five common segments and four bubbles, three of which have two branches. The third bubble is more complicated. The entire graph may be partitioned so that each of its edges lies in a unique line (allowing for degenerate cases, including single edge lines, and circles). (*B*) The same line, but now each bubble has been replaced by a bubble consisting of all its paths. After this change, each bubble consists only of parallel edges.

The remainder of the assembly process consists of a series of operations that modify this graph, so as to improve it. To facilitate these operations, we decompose the graph into units called lines (Fig. 1; Methods). Lines are extended linear regions, punctuated only by “bubbles.” Bubbles are places in the graph where the sequence diverges along alternate paths that then reconnect. Common sources of bubbles are loci that are heterozygous or difficult to read (in particular, at long homopolymers).

We can use lines to scaffold the assembly graph. This involves determining the relative order and orientation of two lines, then breaking the connections at their ends, then inserting a special “gap” edge between the lines. The end result is a new line, which has a special “bubble” consisting only of a gap edge. Subsequent operations (described later) may remove some of these gaps, replacing them by sequence.

Scaffolding is first carried out using read pairs. If the right end of one line is unambiguously connected by read pairs to the left end of another line, then they can be connected. Read pairs can reach over short gaps. To scaffold across larger gaps, we use the barcodes. Briefly, if two lines are actually near each other in the genome, then with high probability, multiple molecules (in the partitions) bridge the gap between the two lines. Therefore for any line, we may find candidate lines in its neighborhood by looking for other lines sharing many of the same barcodes. By scoring alternative orders and orientations (O&Os) of these lines, we can scaffold the lines by choosing their most probable configuration, excluding short lines whose position is uncertain (Methods).

Once the assembly has been scaffolded, some gaps may be replaced by one or more sequences. For short gaps, read pairs from both sides of the gap reach in and may cover the intervening sequence, from which it may be inferred. For long gaps, we first find the barcodes that are incident upon sequence proximate to the left and right sides of the gap. Then we find all the reads in these barcodes. This set of reads will include reads that properly lie within the gap and yet be roughly 10 times larger than that set (as each partition contains about 10 molecules). We assemble this set of reads. Reads outside the gap locus tend to be at low coverage in this restricted read set and hence not assemble. In this way, it is typically possible to fill in the gap with a chunk of graph and thereby remove the gap from the assembly. The chunk may not be a single sequence. For example, at this stage, heterozygous sites within the gap would typically be manifested as simple bubbles.

**Figure 2.**
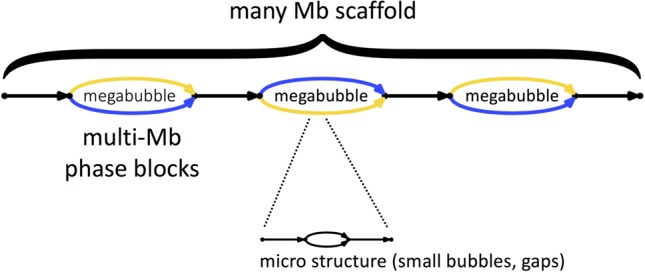
Supernova assemblies encode diploid genome architecture. Each edge represents a sequence. Blue represents one parental allele, and gold represents the other. Megabubble arms represent alternative parental alleles at a given locus, whereas sequences between megabubbles are homozygous (or appear so to Supernova). Successive megabubbles are not phased relative to each other. Smaller scale features appear as gaps and bubbles.

The final step in the assembly process is to phase lines. First for each line (Fig. 1), we find all its simple bubbles, i.e., bubbles having just two branches. Then we define a set of molecules. These are defined by a series of reads from the same barcode, incident upon the line, and not having very large gaps (>100 kb) between successive reads. A given molecule then “votes” at certain bubbles, and the totality of this voting (across all molecules on each line) is then used to identify phaseable sections of the line, which are then separated into “megabubble” arms (Fig. 2; Methods).

### Software and computational performance

Supernova takes as input FASTQ files. No algorithmic parameters are supplied by the user. Supernova is designed to run on a single Linux server. The peak memory usage across the seven human assemblies of this work was 335 GB, and accordingly we recommend using a server having ≥384 GB RAM. Wall clock run times are shown in Table 1 and are in the range of 2 d.

### Supernova output

A Supernova assembly can separate homologous chromosomes over long distances, in this sense capturing the true biology of a diploid genome (Fig. 2). These separated alleles (or phase blocks) are represented as “megabubbles” in the assembly, with each branch representing one parental allele. Sequences between megabubbles are nominally homozygous. Successive megabubbles are not phased relative to each other (if they were, they would have been combined). A chain of megabubbles as shown comprise a scaffold. In addition to large-scale features, the Supernova graph encodes smaller features such as gaps and bubbles at long homopolymers, whose lengths are not fully determined by the data.

**Figure 3.**
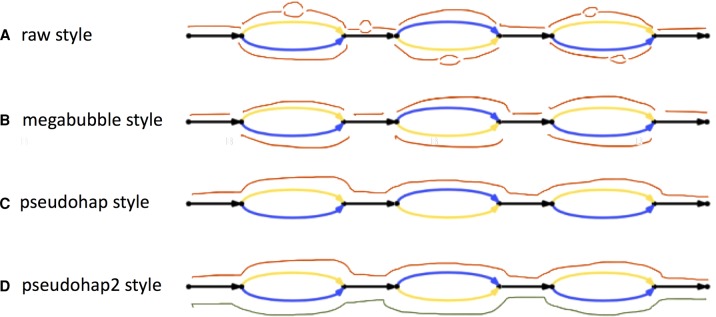
Representation of Supernova assemblies as FASTA. Several styles are depicted. (*A*) The raw style represents every edge in the assembly as a FASTA record (red segments). These include microbubble arms and also gaps (printed as records comprising 100 Ns for gaps bridged by read pairs, or a larger number, the estimated gap size) (Supplemental Note 5). Unresolved cycles are replaced by a path through the cycle, followed by 10 Ns. Bubbles and gaps generally appear once per 10–20 kb; consequently, FASTA records from *A* are much shorter (∼100 times) than those from *B, C,* and *D*. For each edge in the raw graph, there is also an edge written to the FASTA file representing the reverse complement sequence. For the remaining output styles, we flatten each microbubble by selecting the branch having highest coverage, merge gaps with adjacent sequences (leaving Ns), and drop reverse complement edges. (*B*) In this style each megabubble arm corresponds to a FASTA record, as does each intervening sequence. (*C*) The pseudohap style generates a single record per scaffold. As compared to the megabubble style, in the example, seven red edges are seen on *top* (corresponding to seven FASTA records) that are combined into a single FASTA record in the pseudohap style. Megabubble arms are chosen arbitrarily so many records will mix maternal and paternal alleles. (*D*) This style is like the pseudohap option, except that for each scaffold, two “parallel” pseudohaplotypes are created and placed in separate FASTA files.

A Supernova assembly can be translated into FASTA in several distinct ways that might prove useful for different applications (Fig. 3). These allow representation of the full (or “raw”) graph (Fig. 3A), or erase microfeatures (choosing the most likely branch at small bubbles and replacing gap edges by Ns). There is more than one way to package the result, depending on how megabubble branch points are handled (Fig. 3B–D). We note that erasing microfeatures entails some loss of information, as in some cases the wrong branch of a bubble is chosen.

Cycles in the graph provided an interesting test case. By a cycle, we mean a set of one or more edges that include a path from a vertex back to itself. These are left intact in the full graph; however, in the other forms, they are replaced by a path through the cycle that traverses each edge at least once, followed by Ns. This unfortunately signifies a gap (which could in principle represent any sequence), whereas the full graph indicates precisely which sequences could be present at the locus.

### Inferred DNA length

From each of the Supernova assemblies, we inferred the statistics of DNA molecules that were delivered to a partition and thence sequenced. This reflects the quality of input material as well as degradation during the initial steps of library construction. Table 1 shows the inferred values for the length-weighted mean (LWM) of these molecules, as field F. It was in the range of 92–139 kb. The HGP sample was obtained from fresh blood, and yielded the longest DNA. The other samples were obtained from cell lines. The sample NA12878 may have yielded the shortest DNA because of repeated handling of the DNA tube to create multiple libraries, as that DNA sample was used as a control for many experiments (not directly connected to this work).

### Assembly assessment

We assessed in the same way our seven human assemblies and six human assemblies from the literature that represent the state of the art, encompassing a wide range of laboratory approaches, from low coverage (30×) Pacific Biosciences (PacBio) to complex combinations of multiple technologies at much higher coverage (Table 1). These comparison assemblies were downloaded from publicly accessible FTP sites (Supplemental Note 4). For Supernova assemblies, we computed using the pseudohap FASTA output (Fig. 3C), except as noted.

To facilitate a uniform comparison, we computed all statistics from scratch (except as noted), rather than referring to published values. Before computing these statistics, we removed all scaffolds shorter than 10 kb from each assembly, thereby normalizing for differences in the actual cutoffs used in defining the assemblies, which would otherwise significantly affect statistics, including coverage of the genome.

To assess the continuity of the assemblies, we first computed the N50 contig size. The mean across the seven Supernova assemblies was 116 kb, with little variation. The three PacBio-based assemblies had much larger contigs, whereas contigs from the other assemblies were two-fold or more shorter than those from Supernova.

All of the Supernova assemblies were diploid, with N50 phase block size ranging from 2.7 to 9.3 Mb, with variability due presumably to varied ancestry and varied DNA length. Of the six other human assemblies, only the 702× assembly of YH (assembly M) was diploid, and it had an N50 phase block size of 0.5 Mb (as reported). The ∼100 kb molecules underlying Linked-Reads enable the long phase blocks that are difficult to achieve with other technologies.

Scaffolds in the Supernova assemblies ranged from 15.1 to 18.7 Mb (N50). For the PacBio-only assemblies (KL), scaffolds are contigs, as these assemblies have no gaps; these scaffolds are much shorter than the Supernova scaffolds. The four combination assemblies (HIJM) had longer scaffolds, ranging from 23 to 43 Mb. The gappiness (fraction of Ns) in these scaffolds also varied greatly, from 0% for the PacBio-only assemblies, to ∼2% for the Supernova assemblies, to ∼10% for assembly I.

Any assessment of assembly continuity would be tempered by an assessment of the accuracy and completeness of those same assemblies. Although one could do this by comparing to a human reference sequence (and we do so later), the ideal would be to exploit ground truth data from the same sample that was assembled. These data would consist of clones (from individual haplotypes), that had been independently sequenced and assembled, and which were representative of the genome. We could find only two samples, for which such truth data was available and for which high quality DNA could be procured to create assemblies. These were the sample from a living Human Genome Project donor, which we refer to as HGP, for which 340 Mb of finished clones had been sequenced and assembled during the project, at great expense, and NA12878, for which we had previously sequenced and assembled 4 Mb of random clones (Weisenfeld et al. 2014). Although the HGP clones were not truly random, we reasoned that they comprised so much of the genome (∼10%) that they would be reasonably representative of it. They comprise a remarkable and unique resource.

For a given sample, if we knew the exact sequence for each of its chromosomes, we could assess the accuracy of an assembly of that sample by enumerating maximal regions of the genome that are perfectly represented in the assembly. Such regions would be terminated by errors or gaps in the assembly (note that displaying the wrong allele would count as an error). We call the N50 size of such perfectly represented regions the “N50 perfect stretch.” For diploid genomes, if one has both a diploid assembly (thus attempting to display all chromosomes), and representative finished sequence from the exact same sample (thus providing a sample of those chromosomes), then one can approximate the N50 perfect stretch (Supplemental Note 6). There are no publicly available assemblies that satisfy these requirements, other than those generated by Supernova. In particular, we can assess the N50 perfect stretch for Supernova assemblies of HGP and NA12878 in Table 1 (assemblies F and G). These were computed from the raw output (Fig. 3).

**Figure 4.**
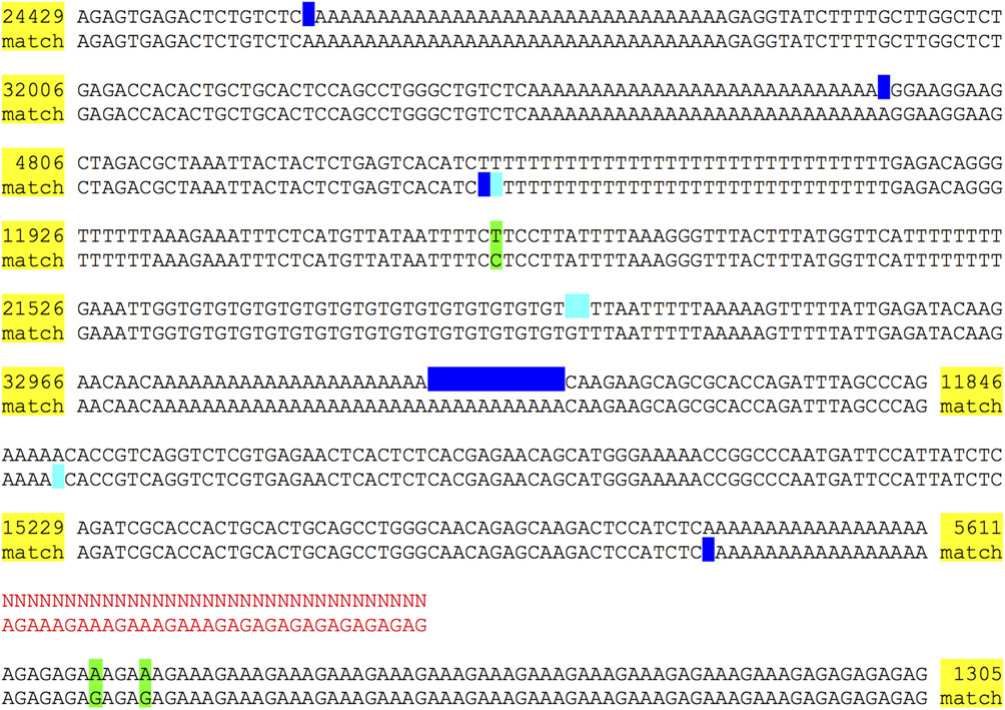
Alignment of Supernova assembly to finished sequence from the same sample. GenBank sequence AC004551.1 for finished clone RPCI1-71H24 has length 162,346 bases, and its reverse complement perfectly matches GRCh37. The clone encompasses a region of Neandertal origin (Mendez et al. 2013). Both the clone and assembly F (Table 1) represent DNA from the same HGP donor. The clone matches a region of which 96% is between two megabubbles in the assembly, thus represented as homozygous. The alignment of the assembly to the clone region on GRCh37 is shown. Each line pair shows the assembly on *top* and the reference on the *bottom*. (Yellow) abbreviated, perfectly matching stretches; (green) mismatched bases; (blue) indels; (cyan) indels, but not present in comparison to raw graph; (red) captured gap: signified by 34 Ns (actual number in assembly is 100); assembly region also has two cycles, each suffixed by 10 Ns in output, not shown. In these cases the flattened sequence for the cycle exactly matches the reference.

We found that the N50 perfect stretch in these Supernova assemblies was 19.8 kb for the HGP assembly and 16.5 kb for the NA12878 assembly (Table 1). The difference might be attributable to sampling error as the finished sequence for NA12878 comprised 4 Mb, whereas the finished sequence for HGP comprised 340 Mb. We further examined the alignments of the finished sequence to the HGP assembly to understand the exact nature of the assembly defects that terminated perfect stretches. For example Figure 4 (and the corresponding alignments for thousands of other clones) show a preponderance of errors in low complexity sequence, particularly, near long homopolymers. These errors might be attributable to library construction defects, sequencing defects, algorithmic defects, or possibly errors in the finished sequence (International Human Genome Sequencing Consortium 2001).

In more detail, Figure 4 displays the alignment of the Supernova HGP assembly to a 162-kb finished clone from the same sample, interesting because it subsumes a region of Neandertal origin (Mendez et al. 2013). Each of several discrepancies between the assembly and finished sequence are annotated. Although we expect that most of these correspond to defects in the Supernova assembly, there is at least one case in which the clone sequence is likely in error: for the first mismatch (shown in green), all reads in the Supernova assembly support the assembly base, and moreover the base was changed in the transition from GRCh37 to GRCh38, so that the latter now matches the assembly.

The exact list of discrepancies depends on the particular form of the Supernova assembly that the clone is compared to (Fig. 4), and this is interesting because it speaks to the power of the different forms. For this clone, only indel discrepancies are affected. If we compare the clone to the raw graph (full assembly), choosing the best path through the graph, then only the indel discrepancies shown in blue occur. However if we instead compare to the pseudohap style, in which small bubbles have been flattened by choosing the most probable path through the bubble (without looking at any reference sequence), then there are additional discrepancies, seen in cyan. Bubbles in the full assembly thus contain some information that is lost in the other forms.

Parental sequence data may also be used to assess the assembly of their child. In particular, this could provide a direct readout on the accuracy of phase blocks in a diploid assembly. This has not been done before for human genomes, because for the two extant diploid human assemblies (Levy et al. 2007; Cao et al. 2015), the parents were not sequenced. For four of the Supernova assemblies, (BCEG in Table 1), the parents had been sequenced, and phased VCFs were available (Supplemental Note 7). This allowed us to estimate the phasing accuracy of these assemblies.

To do this, for each megabubble, whenever we found two positions on alternate megabubble branches that could be mapped to the same position on GRCh37, which represented different bases (a heterozygous SNP), and which was phased in the VCF, we recorded either 0 or 1, depending on whether the “top” branch of the megabubble was assigned to the maternal or paternal allele. A sequence of all 0's or all 1's would represent perfect phasing. We counted all the “votes” (0 or 1), and counted all “wrong votes” (1 if majority = 0, 0 if majority = 1), and summed across all megabubbles of size ≥1 Mb. The global error rate for phasing of a given assembly would be (wrong votes)/votes, noting that a “long switch,” i.e., haplotype misassembly error on even a single megabubble would drive this rate up.

We found that for three individuals (assemblies BCE), the observed phasing error rates were between 0.00006 and 0.00008, whereas for the fourth individual (assembly G), the observed error rate was 0.00077. The difference is attributable to rare “long switch” (haplotype misassembly) events, wherein maternal and paternal sequences are juxtaposed within a megabubble and that just happened to have occurred in assembly G and not the others; for example, exclusion of one such event lowers the rate to 0.00015.

Next, to measure the relative completeness of the different assemblies, we computed the fraction of nonduplicate *k*-mers that were present in the reference sequence (GRCh37) and missing from a given assembly (Supplemental Note 8). We used *k* = 100, balancing between two considerations. First it was important that the fraction of duplicate *k*-mers be small, as the analysis would be blind to them. For *k* = 100, the fraction of duplicate *k*-mers in GRCh37 is 2.3%. Second, we did not want to lose too many *k*-mers to polymorphism. Assuming a polymorphism rate of 1/1000, we would expect ∼10% of *k*-mers would be missing because of bona fide differences between the sample and reference.

Most of the comparison assemblies were haploid, and thus their missing fraction had to be computed based on their haploid output. For the Supernova assemblies, we computed both the haploid missing fraction (based on output type pseudohap) or their diploid missing fraction (based on output type pseudohap2). For assembly M (the 702× diploid assembly of YH), because there was no direct way to divide the assembly into haplotypes, we used the entire assembly and reported only the diploid missing fraction.

For the seven Supernova assemblies, the haploid missing fraction varied from 12.4% to 14.5%, with the highest values for the African samples (as would be expected, assuming that the African samples are most divergent from the reference). In general, the haploid coverage of the comparison assemblies is lower than that for the Supernova assemblies. For example, assembly I is missing 19.7%. The one exception is the 71× PacBio assembly of NA24385 (assembly K), which is missing 11.8%, as compared to the Supernova assembly of the same sample (assembly E), which is missing 13.9%. However, the corresponding diploid Supernova assembly is missing only 9.6%, again lower than the PacBio assembly (which is haploid).

We next analyzed the missing 100-mers by their type (Supplemental Table 2), examining both duplication and GC content, and using diploid assemblies where available. For GC content, we were particularly interested in performance at GC extremes, where we would expect single-molecule technologies to be superior. At low GC (0%–19%), this was not observable. The performance of the Supernova assemblies was actually somewhat better. For example, the total missing *k*-mers in this GC range for the Supernova assembly of NA24385 was 0.144% as compared to 0.156% for the 71× PacBio assembly of the same sample. Conversely at high GC (80%–100%), for the same sample, Supernova missed 0.029%, whereas the PacBio assembly missed 0.019%. Next, we analyzed duplicate *k*-mers, i.e., those occurring more than once in the reference. For these, the PacBio assembly of the same sample had a clear advantage, with 0.127% missing compared with 0.485% for Supernova. Conversely, Supernova excelled at nonduplicate *k*-mers, with 9.127% missing compared with 11.654% for PacBio.

We then assessed long-range accuracy of the assemblies. To do this, for a given assembly and for fixed sizes (1, 10 Mb), we selected all scaffold segments (sequences of the given number of bases, within one FASTA record) of the given size in the assembly, whose end *k*-mers occurred exactly once in the reference sequence. We excluded segments that bridged a gap of size 100 or more in the reference, as these gap sizes could be inaccurate or polymorphic (Bovee et al. 2008). For each segment, we tested its end *k*-mers for consistent placement, meaning lying on the same chromosome, in the correct order and orientation, and defining a fragment whose length is within 10% of the fixed size. The fraction of segments whose end *k*-mers were placed inconsistently is reported in Table 1. Inconsistency could be due to assembly error, large errors in assembly gap measurement, or polymorphism within a sample or between it and the reference.

For the seven Supernova assemblies, and the two distances (1, 10 Mb), all inconsistent fractions were between 0.6% and 2.0%. Two of the comparison assemblies were comparably accurate: assembly H, based on PacBio and BioNano Genomics data; and assembly M (the 702× diploid assembly of YH). The other four comparison assemblies exhibited several-fold higher inconsistency at one or more measurement distances. For example, assembly J (including Dovetail data), had long-range inconsistencies of 5.6% and 6.0%, somewhat qualifying the advantage of its very long scaffolds (42.8 Mb). The 71× PacBio assembly (K) had an inconsistency of 17.9% at 10 Mb, suggesting that PacBio data alone might be insufficient for accurate long-range assembly.

### Replicability of results

To test replicability of the laboratory and computational process for Supernova, we carried out three types of experiments using NA12878, all with ∼38× data sets consisting of 800 M reads sequenced on a HiSeq X instrument (Supplemental Table 3). First, we sequenced the same library at three different HiSeq X sites, thus testing only sequencer-to-sequencer variability. Second, we created five additional libraries and sequenced these on the same flow cell, thus testing only library-to-library variability. For the eight assemblies, some statistics are highly stable, e.g., the N50 contig length varies from 96.5 to 120.0 kb, and the N50 perfect stretch varies from 9.9 to 13.5 kb; whereas other statistics more dependent on rare events are more variable. Thus, the percent inconsistent at 10 Mb varies from 0.5 to 1.6, with that single value over 0.9; similarly, the N50 scaffold size varies from 16.1 to 25.5 Mb. The N75 scaffold size, which is less dependent on rare events, varies less, from 8.7 to 11.4 Mb. Third, we re-ran all eight assemblies a second time, to confirm that identical FASTA output files were generated, which they were.

### Novel sequences in the Supernova assemblies

We assessed the presence of sequences in the Supernova assemblies that were both long and highly divergent from the reference sequence or absent from it entirely. We scanned each diploid assembly for 10-kb windows, for which at most 10% of the 100-mers were contained in GRCh38 (*k*-mers touching gap bases were ignored). Then we merged overlapping windows. Virtually all of these sequences had homology only with primate sequences, with the exception of contaminants, which we removed (Supplemental Note 9). We also filtered out redundant windows (defined by ≥90% 100-mer identity). These would represent novel sequences shared by haplotypes. Supplemental Table 4 describes the statistics of the residual windows. The amount of novel sequence varied from 5.8 to 8.1 Mb, depending on the assembly.

The novel sequences have N50 size 101 kb and vary in length up to 412 kb, but tend to be in relatively small assembly units. For example for HGP, of 8.1 Mb, only 0.4 Mb is present in scaffolds of size ≥10 Mb, or present in scaffolds of size ≥1 Mb, whereas 5.7 Mb is present in scaffolds of size ≥100 kb. The relative lack of continuity in the novel sequence regions suggests that there is substantially more to be found (that would meet the 10-kb threshold).

The fraction of unique 100-mers within a given missing set varied from 71.5% to 80.9%, as expected, given that two alleles might be similar, but not so similar as to have been filtered out. We combined all seven sets of novel sequences and computed the fraction of unique 100-mers, which was 42.7%, suggesting that many new assemblies would be needed to fully characterize “the” human genome. For example, the total number of unique 100-mers in the missing sets for assemblies A–F was 17.8 M, whereas if we add in the one additional assembly (G), we find 19.8 M missing *k*-mers, i.e., 2.1 M more.

## Discussion

Although knowledge of the genome is a fundamental starting point for biology, for large and complex genomes, obtaining that knowledge continues to be a challenge. Low-cost and straightforward methods based on read alignment to a reference provide an extraordinarily valuable but incomplete readout. A far more complete picture can be obtained through complex and sophisticated de novo assembly approaches, but their material requirements and expense preclude widespread use.

Moreover, nearly all de novo assemblies of diploid genomes have been haploid: at each locus they combine together sequences from maternal and paternal chromosomes, yielding as output a single mélange. This both corrupts and loses information; thus, generating diploid assemblies has been a major goal of the field. It has been achieved for genomes up to 5% the size of a human genome (Jones et al. 2004; Chin et al. 2016), and in two cases, at great expense for human genomes (Levy et al. 2007; Cao et al. 2015).

In this work, we demonstrate true diploid human assemblies via a single straightforward library made from ∼1 ng of high molecular weight DNA. We carried out our approach on seven human samples, which we sequenced on the Illumina HiSeq X instrument at low cost. These assemblies used identical code, with identical parameters as a “push-button” process, that ran in 2 d on a single server. The aggregate experiment burden of our approach is dramatically lower than that for all the human assemblies that we compared to. Our approach yields much longer phase blocks than the previous diploid human assemblies (Levy et al. 2007; Cao et al. 2015). Our diploid human assemblies are the first to be validated using finished sequence from the same sample and the first whose phasing accuracy has been validated using parental sequences.

We anticipate utility of our new method both for routine use as a single-technology approach and in combination with other technologies, e.g., for “The Reference Genomes Improvement” project (Steinberg et al. 2016). We have demonstrated our method here only on human genomes, and we are confident that it will work well on similar genomes, for example, most mammals. Fundamentally different genomes (including much smaller ones, as well as polyploid genomes) will likely require modifications to our methods. For smaller genomes, the primary goal is to achieve the same level of molecular coverage (LPM = links per molecule) that is now achieved for human genomes. The most direct approach would be to simultaneously reduce the input mass loaded, increase the sequencing depth of coverage, and subsample the reads by barcode.

Our diploid assemblies open the door to new analytical approaches, including alignment of assemblies to a reference sequence to call variants. The low cost and burden of our approach makes it applicable to large-scale projects, both for human and “new” genomes, posing new opportunities and challenges both for experimental design and biological interpretation.

## Methods

### Genomic DNA samples

Peripheral blood samples were obtained from a donor (anonymous, although known to 10x Genomics, and labeled HGP in Table 1). The study was performed under written informed consent from the donor and approved by the 10x legal department. Genomic DNA (gDNA) was extracted from fresh whole blood according to 10x Sample Preparation Demonstrated Protocol “DNA Extraction from Whole Blood” (http://support.10xgenomics.com/de-novo-assembly/sample-prep/doc/demonstrated-protocol-hmw-dna-extraction-from-whole-blood). Optimal performance has been characterized on high molecular weight (HMW) gDNA with a mean length >50 kb. The gDNA did not require further size selection or processing.

Commercially acquired samples were obtained (NA12878, NA19238, NA19240, HG00733, HG00512, NA24385, and NA24143 from Coriell). Genomic DNA was extracted from human lymphocyte cells following the HMW gDNA extraction protocol outlined in the Chromium Genome User Guide Rev A (http://support.10xgenomics.com/de-novo-assembly/sample-prep/doc/user-guide-chromiumtm-genome-reagent-kit).

Genomic DNA was quantified with the Qubit dsDNA HS Assay Kit (Life Technologies) according to 10x Sample Preparation Demonstrated Protocol “High Molecular Weight DNA QC” (https://support.10xgenomics.com/de-novo-assembly/sample-prep/doc/user-guide-chromium-genome-reagent-kit-v1-chemistry).

### Sequencing library construction using the Chromium Genome Reagent Kit

A Chromium Controller Instrument (10x Genomics) was used for sample preparation. The platform allows for the construction of eight sequencing libraries by a single person in 2 d. Sample indexing and partition barcoded libraries were prepared using the Chromium Genome Reagent Kit (10x Genomics) according to manufacturer's protocols described in the Chromium Genome User Guide Rev A (https://support.10xgenomics.com/de-novo-assembly/sample-prep/doc/user-guide-chromium-genome-reagent-kit-v1-chemistry). Briefly, in the microfluidic Genome Chip, a library of Genome Gel Beads was combined with an optimal amount of HMW template gDNA in Master Mix and partitioning oil to create GEMs. Template gDNA (1.25 ng) was partitioned across approximately 1 million GEMs, with the exception of the peripheral blood sample, which utilized 1 ng of template gDNA. Upon dissolution of the Genome Gel Bead in the GEM, primers containing (1) an lllumina R1 sequence (Read 1 sequencing primer), (2) a 16-bp 10x Barcode, and (3) a 6-bp random primer sequence were released. GEM reactions were isothermally incubated (for 3 h at 30°C ; for 10 min at 65°C; held at 4°C), and barcoded fragments ranging from a few to several hundred base pairs were generated. After incubation, the GEMs were broken and the barcoded DNA was recovered. Silane and Solid Phase Reversible Immobilization (SPRI) beads were used to purify and size select the fragments for library preparation.

Standard library prep was performed according to the manufacturer's instructions described in the Chromium Genome User Guide Rev A (https://support.10xgenomics.com/de-novo-assembly/sample-prep/doc/user-guide-chromium-genome-reagent-kit-v1-chemistry) to construct sample-indexed libraries using 10x Genomics adaptors. The final libraries contained the P5 and P7 primers used in lllumina bridge amplification. The barcode sequencing libraries were then quantified by qPCR (KAPA Biosystems Library Quantification Kit for Illumina platforms). Sequencing was conducted with an Illumina HiSeq X with 2×150 paired-end reads based on the manufacturer's protocols.

### Internal representation of Supernova assemblies

An assembly is first represented as a directed graph in which each edge represents a single strand of DNA sequence. Abutting edges overlap by *k*-1 bases (*k* = 48). This graph is called the base graph. Subsequently, a new graph called the super graph is constructed in which each edge represents a path in the base graph. Each super-graph edge may be translated into a DNA sequence. Where super-graph edges abut, their associated sequences overlap by *k*−1 bases (*k* = 48, the same as the base graph). An advantage of this approach was that it allowed for certain computations to be carried out on the base graph, once, so that experiments involving super-graph algorithm improvement could be carried out, without repeating those computations. It is also a convenient mechanism for effectively changing the *k*-value, without changing the actual *k*-value in use.

### Generation of the initial super graph

For each read pair, where possible, we find one (or sometimes more) paths in the graph that could represent the sequence of the originating insert (Weisenfeld et al. 2014). These paths are represented as sequences of integers corresponding to the identifiers of edges in the base graph. Whenever there are two paths that perfectly overlap by *k*′ = 200 bases, we formally join them via an equivalence relation. This yields the super graph.

### Definition of lines

A line in a graph is an acyclic subgraph, having a unique source and sink (relative to the subgraph), with one edge exiting the source and one edge entering the sink (and maximal with respect to these properties). We also allow for the special case of circles, which are similar, but have no source or sink. Every edge lies in a unique line.

### Ordering and orienting lines using barcodes

For all lines in the assembly, we carry out an initial computation, which assigns a linear coordinate system to each line and marks the positions of uniquely placed reads on it, organized by barcode. Now for a given line set S, we score alternative O&O possibilities. Each O&O for S thus yields a sequence of barcoded read positions along a hypothetical merged line. We compute a penalty for the given O&O, which is a sum, over all its constituent barcodes. For each barcode, we first compute the mean separation between successive read placements for that barcode (in the merged line). Then we traverse these placements, in order, finding those pairs of consecutive placements that bridge a jump from one constituent line to another, and which may thus represent a misconnection. We divide the separation for this pair by the mean separation for the barcode. If the quotient is smaller than a fixed bound (2.0), we discard it on the theory that it is unlikely to represent an anomaly. The remaining quotient is added to the penalty (Supplemental Fig. 1). A given O&O is treated as the “winner” if its penalty is at least a fixed heuristic amount (60.0) less than that for competing tested O&O possibilities for the same set of lines.

### Phasing

A “phasing” is an orientation of each bubble on a line, placing one of its branches on “top” and the other on the “bottom.” Initially we choose an arbitrary orientation for the bubbles. Each molecule touches some bubbles, and thus (relative to a given phasing) may be represented as a sequence with entries +1 for top, −1 for bottom, or 0 for silent. A phasing is “good” if each molecule is coherent, containing nearly all 1's or nearly all −1's (plus 0's at silent positions). Accordingly, we define the score of a phasing to be the sum over all molecules of Max(pluses,minuses) − Min(pluses,minuses).

We then carry out iterative perturbations, each of which flips some bubbles, and keeping only those perturbations that increase the phasing score. Three types of perturbations are attempted:

1. We flip bubbles on a given molecule to make it completely coherent.
2. We flip an individual bubble.
3. We pivot at a given point, flipping all bubbles to its left.

This yields an initial phasing. We then look for weaknesses in it. First, if flipping a bubble has too small an effect on the score, we exclude it from the phasing operation. For example, a bubble might arise at a long homopolymer, whose length was fixed in the sample but changed during data generation. These uncertain bubbles are “copied” to both megabubble branches. Second, if a pivot has too small an effect on the score, we break the phasing at the pivot point, yielding multiple phase blocks for the given scaffold. For example this could happen if a sufficiently long region in a given sample was homozygous.

## Data access

Raw sequence data from this study have been submitted to the NCBI Sequence Read Archive (SRA; http://www.ncbi.nlm.nih.gov/sra) under accession number SRP090941. Assemblies have been deposited at DDBJ/ENA/GenBank (https://www.ncbi.nlm.gov/genbank/) under the accessions MSBx000 00000 where x is in {J,K,L,M,N,O,P}, and the versions described in this work are MSBx01000000. A file of 3431 finished clones from GenBank for the HGP sample, which we used for assessment, is available as Supplemental Material (HGP.GenBank.fasta.gz). These materials are also available at http://support.10xgenomics.com/de-novo-assembly/datasets. Source code is available as Supplemental Material (supernova-1.1-source.tar.gz) and also at https://github.com/10XGenomics/supernova. In addition, creation of a cell line from the HGP donor is in progress (Coriell #GM26200).

## Competing interest statement

All authors are employees and stockholders of 10x Genomics.

## Supplementary Material

Supplemental Material
