# Supplementary material for: Direct determination of diploid genome sequences
Source: Genome Res. 2017 May;27(5):757–67. doi: 10.1101/gr.214874.116 (PMC5411770; doi:10.1101/gr.214874.116)
Supplement: Supplemental Material [file supp_27_5_757__index.html]

Direct determination of diploid genome sequences — Direct determination of diploid genome sequences — Supplemental Material 

# Direct determination of diploid genome sequences

## Supplemental Material

- Supplemental\_Table\_S1.docx
- Supplemental\_Table\_S2.docx
- Supplemental\_Table\_S3.docx
- Supplemental\_Table\_S4.docx
- Supplemental\_Fig\_S1.pdf
- Supplemental\_Fig\_S2.pdf
- Supplemental\_supernova-1.1-source.tar.gz
- Supplemental\_HGP\_GenBank.fasta.gz
