# Supplementary material for: Direct determination of diploid genome sequences
Source: Genome Res. 2017 May;27(5):757–67. doi: 10.1101/gr.214874.116 (PMC5411770; doi:10.1101/gr.214874.116)
Supplement: Supplemental Material [file supp_gr.214874.116_Supplemental_Table_S1.docx]

**Supplemental Table 1. Inferred molecule length statistics for Supernova samples**

| **id** | **sample** | **length-weighted mean (kb)** | **mean (kb)** | **std. dev. (kb)** | **median (kb)** |
| --- | --- | --- | --- | --- | --- |
| A | NA19238 | 115 | 59 | 57 | 40 |
| B | NA19240 | 125 | 64 | 62 | 45 |
| C | HG00733 | 106 | 57 | 53 | 40 |
| D | HG00512 | 102 | 58 | 51 | 42 |
| E | NA24385 | 120 | 60 | 60 | 41 |
| F | HGP | 139 | 81 | 69 | 57 |
| G | NA12878 | 092 | 51 | 46 | 36 |

**Supplemental Table 1. Inferred molecule length statistics for Supernova samples.** Statistics about the molecule size distribution in Supernova libraries, supplementing **Table 1**. The length-weighted mean column duplicates column F from **Table 1**.
