# Supplementary material for: Direct determination of diploid genome sequences
Source: Genome Res. 2017 May;27(5):757–67. doi: 10.1101/gr.214874.116 (PMC5411770; doi:10.1101/gr.214874.116)
Supplement: Supplemental Material [file supp_gr.214874.116_Supplemental_Table_S2.docx]

**Supplemental Table 2. Analysis of missing *k*-mers**

| **input** | | | | | | **missing *k*-mers (%)** | | | | | | |
| --- | --- | --- | --- | --- | --- | --- | --- | --- | --- | --- | --- | --- |
| **id** | **sample** | **ethnicity** | **sex** | **data description** | **X** | **by GC content** | | | | | **by duplication** | |
|  |  |  |  |  |  | **0-19** | **20-39** | **40-59** | **60-79** | **80-100** | **dup** | **nondup** |
| A | NA19238 | Yoruban | F | one 10x library | 56 | 0.152 | 4.369 | 4.777 | 0.712 | 0.031 | 0.438 | 9.604 |
| B | NA19240 | Yoruban | F | one 10x library | 56 | 0.152 | 4.328 | 4.658 | 0.684 | 0.026 | 0.443 | 9.406 |
| C | HG00733 | Puerto Rican | F | one 10x library | 56 | 0.139 | 4.042 | 4.376 | 0.655 | 0.030 | 0.418 | 8.823 |
| D | HG00512 | Chinese | M | one 10x library | 56 | 0.150 | 4.416 | 4.701 | 0.686 | 0.030 | 0.480 | 9.503 |
| E | NA24385 | Ashkenazi | M | one 10x library | 56 | 0.144 | 4.233 | 4.533 | 0.673 | 0.029 | 0.485 | 9.127 |
| F | HGP | European | M | one 10x library | 56 | 0.148 | 3.936 | 4.121 | 0.590 | 0.025 | 0.475 | 8.345 |
| G | NA12878 | European | F | one 10x library | 56 | 0.149 | 4.047 | 4.286 | 0.612 | 0.024 | 0.418 | 8.700 |
| H | NA12878 | European | F | unknown number of PacBio libraries plus BioNano Genomics data | 46 | 0.162 | 6.734 | 9.372 | 1.674 | 0.059 | 0.245 | 17.756 |
| I | NA12878 | European | F | 6 libraries (fragment, jumping, 10x) | 160 | 0.212 | 7.760 | 10.097 | 1.525 | 0.089 | 0.813 | 18.870 |
| J | NA12878 | European | F | 9 libraries (fragment, jumping, Fosmid, Chicago) | 150 | 0.176 | 6.512 | 6.939 | 1.059 | 0.083 | 0.662 | 14.108 |
| K | NA24385 | Ashkenazi | M | 7 PacBio libraries | 71 | 0.156 | 5.483 | 5.477 | 0.646 | 0.019 | 0.127 | 11.654 |
| L | NA24143 | Ashkenazi | F | 2 PacBio libraries | 30 | 0.176 | 6.545 | 6.643 | 0.867 | 0.026 | 0.248 | 14.010 |
| M | YH | Chinese | M | ~18,000 Fosmid pools and 6 fragment and jumping libraries, Illumina sequenced, plus Complete Genomics data | 702 | 0.179 | 4.523 | 4.861 | 0.805 | 0.070 | 0.414 | 10.025 |

**Supplemental Table 2. Analysis of missing *k*-mers.** Input description: same as in **Table 1**. Missing *k*-mers: same as in **Table 1**, k = 100, now broken into categories by GC content, and broken into categories by duplicate (‘dup’) or nonduplicate (‘nondup’). Duplicate *k*-mers are those that appear more than once in GRCh37. GC columns add up to the total missing *k*-mer percent, as do duplication columns. We used diploid assemblies wherever available. The fraction of GRCh37 *k*-mers in the five GC bins is 0.7%, 48.1%, 46.4%, 4.7% and 0.1%, respectively. The fraction of GRCh37 *k*-mers that are duplicated is 0.9%.
