# Supplementary material for: Direct determination of diploid genome sequences
Source: Genome Res. 2017 May;27(5):757–67. doi: 10.1101/gr.214874.116 (PMC5411770; doi:10.1101/gr.214874.116)
Supplement: Supplemental Material [file supp_gr.214874.116_Supplemental_Table_S3.docx]

**Supplemental Table 3. Technical replicates**

| **input** | | | **continuity** | | | | | **comparison to truth data** | | | | | |
| --- | --- | --- | --- | --- | --- | --- | --- | --- | --- | --- | --- | --- | --- |
|  |  |  |  |  |  |  |  | **same** | **parental** | **reference** | | | |
| **id** | **library** | **sequencing site** | **N50 contig (kb)** | **N50 phase block (Mb)** | **N50 scaffold (Mb)** | **N75 scaffold (Mb)** | **gappi-**  **ness** | **N50 perfect stretch (kb)** | **phasing error rate** | **missing *k*-mers (%)** | | **inconsistent at given distance (%)** | |
|  |  |  |  |  |  |  |  |  |  | **haploid** | **diploid** | **1 Mb** | **10 Mb** |
| 1 | 1 | Hudson-Alpha | 104.9 | 1.5 | 22.5 | 10.8 | 2.6 | 10.8 | 0.02707 | 13.0 | 10.1 | 1.0 | 0.6 |
| 2 | 1 | GeneWhiz | 101.7 | 1.5 | 25.5 | 11.4 | 2.8 | 11.8 | 0.01009 | 13.1 | 10.2 | 1.0 | 0.6 |
| 3 | 1 | Macrogen | 120.0 | 1.7 | 20.0 | 9.6 | 2.2 | 13.5 | 0.00415 | 12.7 | 9.7 | 1.1 | 0.5 |
| 4 | 2 | Hudson-Alpha | 104.1 | 1.5 | 18.0 | 8.9 | 3.0 | 10.6 | 0.01583 | 13.1 | 10.2 | 1.0 | 1.6 |
| 5 | 3 | Hudson-Alpha | 96.5 | 1.4 | 20.2 | 9.8 | 3.4 | 10.6 | 0.00333 | 13.5 | 10.7 | 1.1 | 0.6 |
| 6 | 4 | Hudson-Alpha | 98.5 | 1.4 | 24.7 | 11.3 | 3.4 | 10.4 | 0.01707 | 13.5 | 10.6 | 1.2 | 0.8 |
| 7 | 5 | Hudson-Alpha | 101.7 | 1.5 | 16.1 | 8.7 | 3.1 | 10.3 | 0.00377 | 13.2 | 10.4 | 1.1 | 0.6 |
| 8 | 6 | Hudson-Alpha | 100.1 | 1.4 | 18.1 | 9.1 | 3.4 | 9.9 | 0.00388 | 13.5 | 10.6 | 1.2 | 0.9 |

**Supplemental Table 3. Technical replicates.** Sixteen assemblies were carried out using 800M reads (38x) from NA12878, all sequenced on HiSeq X instruments. Six libraries were created and sequenced. One library was sequenced at three different sites. (See **Supplemental Note 1**: these are the same data.) After the eight datasets were assembled, we assembled them all a second time, verifying that results were identical. Assembly statistics are as in **Table 1**. We added the N75 scaffold size.
