# Supplementary material for: Direct determination of diploid genome sequences
Source: Genome Res. 2017 May;27(5):757–67. doi: 10.1101/gr.214874.116 (PMC5411770; doi:10.1101/gr.214874.116)
Supplement: Supplemental Material [file supp_gr.214874.116_Supplemental_Table_S4.docx]

**Supplemental Table 4. Novel sequences in Supernova human assemblies**

| **sample information** | | | | **missing sequences** | | |
| --- | --- | --- | --- | --- | --- | --- |
| **id** | **sample** | **ethnicity** | **sex** | **count** | **total (Mb)** | **% unique** |
| A | NA19238 | Yoruban | F | 156 | 6.4 | 71.5 |
| B | NA19240 | Yoruban | F | 147 | 7.0 | 73.9 |
| C | HG00733 | Puerto Rican | F | 122 | 6.3 | 76.5 |
| D | HG00512 | Chinese | M | 122 | 6.2 | 72.6 |
| E | NA24385 | Askenazi | M | 131 | 5.8 | 80.9 |
| F | HGP | European | M | 159 | 8.1 | 75.6 |
| G | NA12878 | European | F | 138 | 6.7 | 77.0 |

**Supplemental Table 4. Novel sequences in Supernova human assemblies.** Sample information: as in **Table 1**. Missing sequences: described in text. Count: number of novel sequences. Total: total size of sequences in Mb. Percent unique: fraction of 100-mers in a given missing set that remain after removing 100-mers that appear two or more times within the set.
