# Supplementary material for: Direct determination of diploid genome sequences
Source: Genome Res. 2017 May;27(5):757–67. doi: 10.1101/gr.214874.116 (PMC5411770; doi:10.1101/gr.214874.116)
Supplement: Supplemental Material [file supp_gr.214874.116_Supplemental_Fig_S1.pdf]

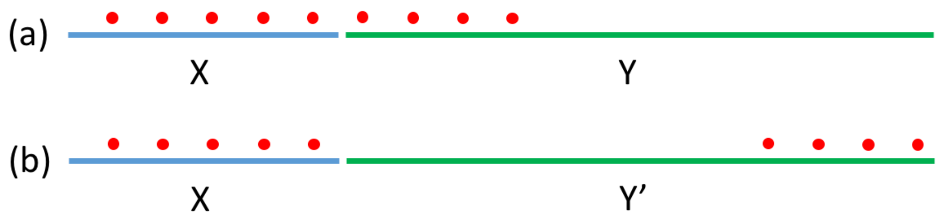

Supplemental Figure 1. Scoring of alternative orders and orientations. Red dots represent reads from one barcode. Lines X and Y are depicted. (a) Correct order and orientation: X, then Y. The two reads abutting the juncture of X and Y have separation 1, as compared to the mean separation for all consecutive reads from the barcode, also 1. The quotient 1/1 = 1 is less than a fixed threshold 2, and thus does not contribute to the penalty for XY. (b) Incorrect orientation: X, then the reverse complement Y’ of Y. The two reads abutting the juncture of X and Y’ have separation 9, as compared to the mean separation (1+1+1+1+9+1+1+1)/8 = 2. The quotient 9/2 = 4.5 exceeds the fixed threshold 2 and thus contributes 4.5 to the penalty for XY’.
