# Supplementary material for: Direct determination of diploid genome sequences
Source: Genome Res. 2017 May;27(5):757–67. doi: 10.1101/gr.214874.116 (PMC5411770; doi:10.1101/gr.214874.116)
Supplement: Supplemental Material [file supp_gr.214874.116_Supplemental_Fig_S2.pdf]

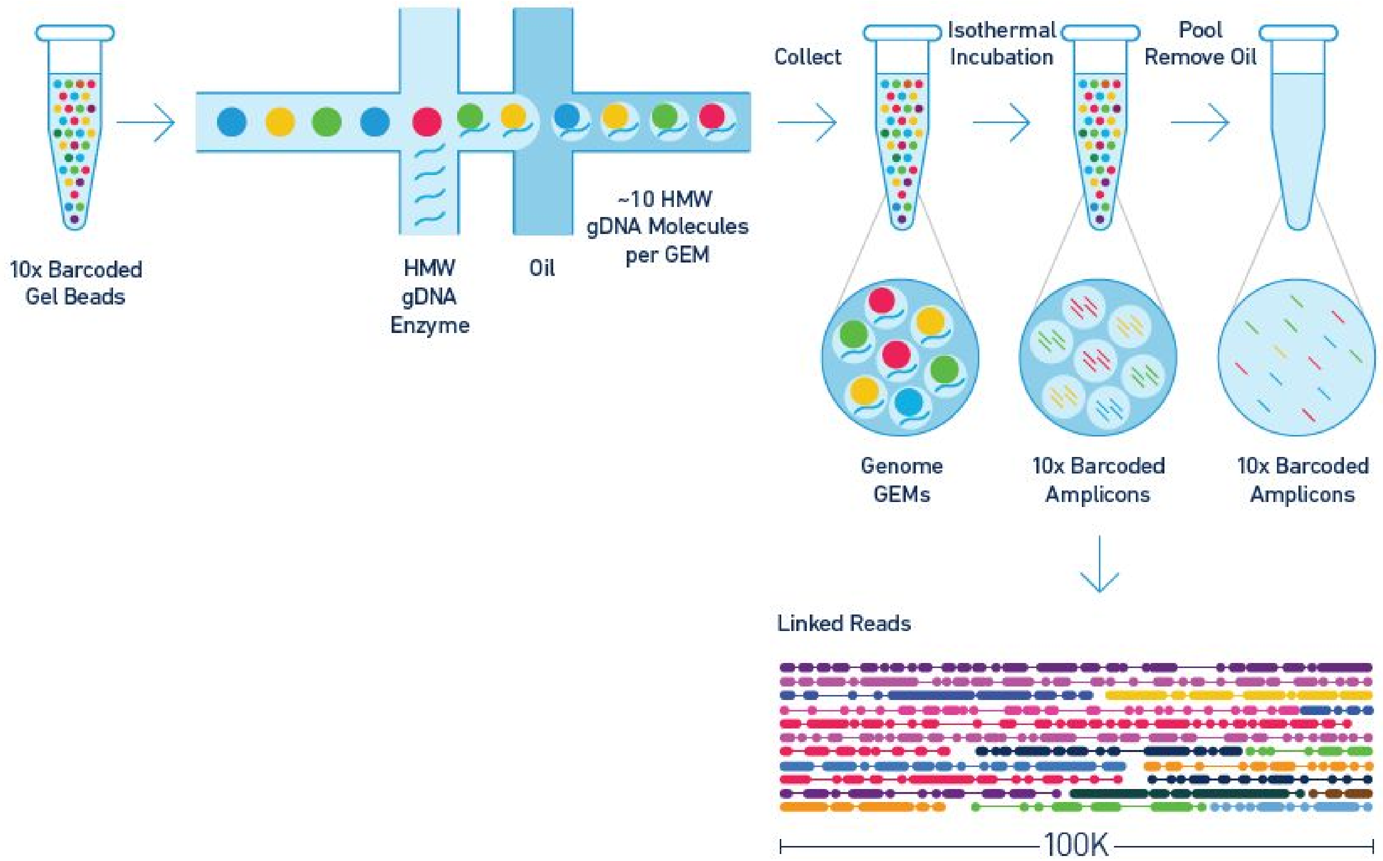

Supplemental Figure 2. Generation of Linked-Reads. An automated microfluidic system allows for functionalized gel beads to be combined with high molecular weight DNA (HMW gDNA) and oil to form a 'Gel Bead in Emulsion (GEM)'. Each GEM contains ~10 molecules of HMW gDNA and primers with unique barcodes. Isothermal incubation allows for the addition of a unique barcode to all DNA within the GEM, and barcoded fragments (typically ~350 bp) are generated. These fragments are then sequenced on an Illumina instrument, yielding groups of reads, organized by barcode, called Linked-Reads.
